# The molecular conformation of silk fibroin regulates osteogenic cell behavior by modulating the stability of the adsorbed protein-material interface

**DOI:** 10.1038/s41413-020-00130-0

**Published:** 2021-02-11

**Authors:** Yanlin Long, Xian Cheng, John A. Jansen, Sander G. C. Leeuwenburgh, Jing Mao, Fang Yang, Lili Chen

**Affiliations:** 1 Department of Stomatology, Union Hospital, Tongji Medical College, Huazhong University of Science and Technology, Wuhan, 430022 China; 2 Hubei Province Key Laboratory of Oral and Maxillofacial Development and Regeneration, Wuhan, 430022 China; 3 Department of Dentistry–Biomaterials, Radboud University Medical Center, Philips van Leydenlaan 25, 6525 EX Nijmegen, The Netherlands; 4 Center of Stomatology, Tongji Hospital, Tongji Medical College, Huazhong University of Science and Technology, Wuhan, 430030 China

**Keywords:** Bone, Calcium and vitamin D

## Abstract

Silk fibroin (SF) can be used to construct various stiff material interfaces to support bone formation. An essential preparatory step is to partially transform SF molecules from random coils to β-sheets to render the material water insoluble. However, the influence of the SF conformation on osteogenic cell behavior at the material interface remains unknown. Herein, three stiff SF substrates were prepared by varying the β-sheet content (high, medium, and low). The substrates had a comparable chemical composition, surface topography, and wettability. When adsorbed fibronectin was used as a model cellular adhesive protein, the stability of the adsorbed protein-material interface, in terms of the surface stability of the SF substrates and the accompanying fibronectin detachment resistance, increased with the increasing β-sheet content of the SF substrates. Furthermore, (i) larger areas of cytoskeleton-associated focal adhesions, (ii) higher orders of cytoskeletal organization and (iii) more elongated cell spreading were observed for bone marrow-derived mesenchymal stromal cells (BMSCs) cultured on SF substrates with high vs. low β-sheet contents, along with enhanced nuclear translocation and activation of YAP/TAZ and RUNX2. Consequently, osteogenic differentiation of BMSCs was stimulated on high β-sheet substrates. These results indicated that the β-sheet content influences osteogenic differentiation of BMSCs on SF materials in vitro by modulating the stability of the adsorbed protein-material interface, which proceeds via protein-focal adhesion-cytoskeleton links and subsequent intracellular mechanotransduction. Our findings emphasize the role of the stability of the adsorbed protein-material interface in cellular mechanotransduction and the perception of stiff SF substrates with different β-sheet contents, which should not be overlooked when engineering stiff biomaterials.

## Introduction

Silk fibroin (SF), a natural protein derived from *Bombyx mori* silk cocoons, is a millennium-old material that has been recently widely adopted in biomedical engineering.^1^ SF exhibits attractive features for the production of bone-related biomaterials, such as robust mechanical properties,^2^ hypoallergenic features,^3^ vascularization,^4^ tunable biodegradation,^5^ the ability to accelerate biomineralization of collagen,^6^ and the ability to act as template for the growth of hydroxyapatite.^7^ Over the past decades, SF has been shown to be a promising polymer to construct various stiff materials to support bone formation. The application of stiff SF materials ranges from scaffolds^1^ to implants,^2^ membranes,^7,8^ and coatings.^3,9^

Regardless of the forms, stiff SF materials are prepared from SF aqueous solutions extracted and regenerated from silk cocoons, which consist of SF molecules in a soluble random coil conformation.^3^ An essential step during the preparation is to partially transform the SF molecules from a random coil to stable β-sheet conformation to render the materials water-insoluble.^10^ The efficiency of β-sheet transformation highly depends on the applied processing methods and has been reported to be ~15%–60%.^10–12^ In previous studies, various groups observed variation with respect to the in vitro osteogenic performance of stiff SF materials with different β-sheet contents.^13–16^ Although these studies indicated that this variation might be partially due to a difference in SF conformation, they failed to exclude the role of other influential factors on cells, such as chemical composition or surface topography (e.g., deformation during the preparatory process). The influence of SF conformation on in vitro osteogenic cell behavior at the material surface is still unclear.

The β-sheet content determines the density of the water-insoluble molecular network of the β-sheets in stiff SF materials. The different β-sheet contents can induce a clear difference in dissolution (degradation) profiles,^17,18^ which might have a strong influence on the surface stability of SF materials. However, SF molecules themselves do not contain recognition motifs, such as integrin-binding arginyl-glycyl-aspartic acid motifs, to facilitate cell adhesion and spreading.^13^ Instead, the adsorbed cellular adhesive protein layer, such as fibronectin (FN), at the SF material surface acts as a bridge between cells and material, where the interfacial stability (e.g., conformation change, reorganization, and detachment) of protein plays an important role in regulating cell behavior.^19^ Focal adhesion (FA) is cell-substrate contact, which forms a link with the adsorbed proteins and the cytoskeleton of spreading cells at the cell-material interface.^20^ Once adhered, cells will generate tensions via the cytoskeleton, and the generated contractility will be sequentially transmitted to the protein layer via FAs to partially unfold, reorganize, or even detach proteins from the underlying material substrate.^21^ In turn, the interfacial instability, especially the detachment of the adsorbed proteinaceous layer induced by cells, has been found to largely disturb intracellular tension via the cytoskeleton.^22^ Consequently, the adsorbed protein-FA-cytoskeleton link plays an important role in the transmission of extracellular cues from the material to the cell and evokes intracellular changes in cytoskeletal organization, which affects subsequent cell funcations.^23^

Yes-associated protein/transcriptional coactivator with PDZ-binding motif (YAP/TAZ) was recently identified as a master regulator in cellular sensing and transduction of mechanical signals, with enhanced nuclear translocation in response to increased intracellular tension.^24–26^ YAP/TAZ has been demonstrated to modulate a wide variety of material-mediated mechanotransduction parameters, such as topography,^27^ degradation,^28^ stress relaxation,^29^ fiber density,^30^ and multicyclic attachment/detachment of cells.^31^ YAP and TAZ are found both in the cytoplasm and nucleus, and they can interact with and activate a number of their DNA binding partners (e.g., Runt-related transcription factor 2 (RUNX2)^30–32^) in the nucleus to modulate the osteogenic differentiation of stem cells. Therefore, the subcellular localizations of YAP/TAZ and RUNX2 have been considered rapid and reliable proxies applied to evaluate material-mediated mechanotransduction by cytoskeletal cues.^25,32^

Therefore, this study aimed to investigate the impact of the β-sheet content of stiff SF materials on osteogenic cell behavior while focusing on the stability of adsorbed protein-material interfaces, adsorbed protein-FA-cytoskeleton link and YAP/TAZ regulation. To shed light on this regulation, we established a material interface model by constructing three stiff SF material substrates with high (SFH group), medium (SFM group), and low (SFL group) contents of β-sheets while maintaining comparable chemical composition, surface topography, and wettability. The surface stability of the SF substrates was assessed using an artificial external force. FN, a ubiquitous cellular adhesive protein, has been applied as a model protein in many studies to evaluate cellular adhesive protein adsorption and stability on materials.^19,33,34^ We also used FN as a model protein to investigate adsorbed protein detachment resistance with the same artificial external force or in a cell culture environment. Subsequently, the number of cytoskeleton-associated FAs, cytoskeletal organization and spreading shape of bone marrow-derived mesenchymal stromal cell (BMSC) response to the different SF substrates were assessed. Finally, the intracellular mechanotransduction of YAP/TAZ and RUNX2 and the consequent osteogenic differentiation of BMSCs on different SF substrates were evaluated.

## Results

### Material characterization of SF substrates with different β-sheet contents

**Fig. 1:**
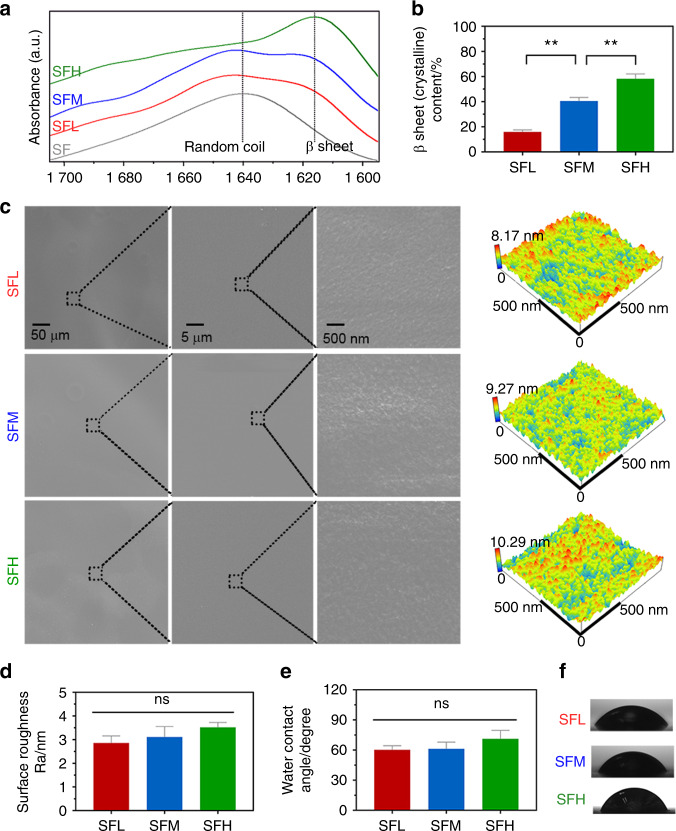
Material characterization of SF substrates with different β-sheet contents. **a, b** FTIR absorbance spectra (**a**) of the amide I region (between 1 695 and 1 595 cm^−1^) obtained from different SF substrates and (**b**) the β-sheet contents calculated by Fourier self-deconvolution from these spectra. **c** Surface topography observed by SEM and AFM. **d** Surface roughness values analyzed by AFM. **e, f** Surface wettability (**e**) determined by water contact angle measurement and (**f**) representative images of water droplets. Error bars represent one standard deviation. (*P < 0.05 and **P < 0.01)

Three SF substrates were constructed with either low, medium, or high β-sheet contents of 15.8% (SFL group), 40.4% (SFM group), and 58.2% (SFH group), respectively (Fig. 1a, b). Ultraviolet sterilization did not significantly influence the SF conformation (Supplementary Fig. 1).

Scanning electron microscopy (SEM) and atomic force microscopy (AFM) images (Fig. 1c) showed that the surface topography of the SF substrates was equally even and smooth regardless of the β-sheet content. The average values of roughness (Ra) measured by AFM (Fig. 1d) did not show any significant difference among the groups (*P* > 0.05).

The average water contact angle slightly increased with increasing β-sheet content (Fig. 1e, f). Moreover, this factor was not significantly influenced by ultraviolet sterilization (Supplementary Fig. 2). With increasing β-sheet content, the stiffness of the SF substrates increased from 14.8 ± 4.1 MPa to 120.3 ± 17.4 MPa (Supplementary Table 1).

### Stability of material-protein interfaces with different β-sheet contents

**Fig. 2:**
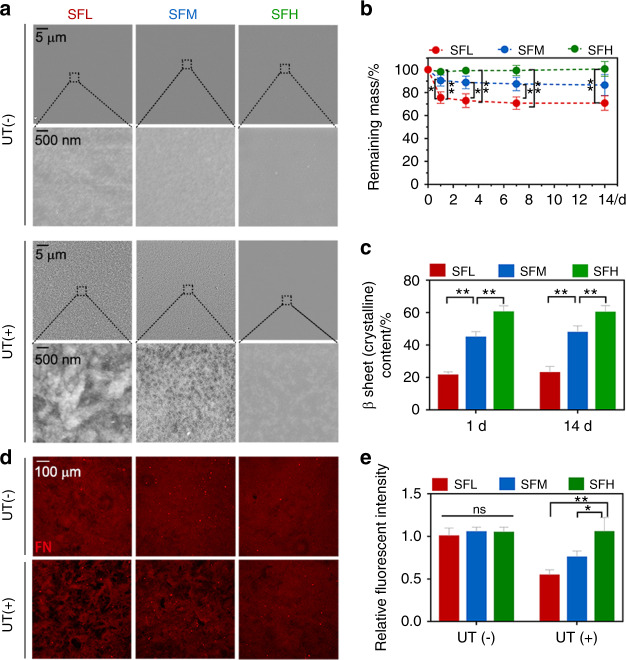
Interfacial stability between different SF substrates and adsorbed FN. **a** SEM images of different SF substrates after being immersed in PBS for 24 h, with and without ultrasonic treatment (UT). **b, c** Remaining mass (**b**) and β-sheet contents (**c**) of different SF substrates immersed in PBS with UT treatment at specific time points. **d, e** Immunofluorescence images (**d**) and the quantitative analysis (**e**) of adsorbed FN on different SF substrates after being immersed in FN solution for 24 h, with and without UT treatment. Error bars represent one standard deviation. (*P < 0.05 and **P < 0.01)

After being immersed in phosphate-buffered saline (PBS) for 24 h, the samples were removed and gently rinsed with Milli-Q water. The topography of the different material surfaces was comparable among the three groups (Fig. 2a). However, when a mild ultrasonic treatment was applied, the surface topography of the SFL substrate became much more uneven and rougher than that of the SFM and SFH substrates.

Quantitative measurements were performed in terms of the remaining mass of the substrates with the same ultrasonic treatment (Fig. 2b). The results showed a 24.5% weight loss on the SFL surfaces after the first removal of loosened components at 24 h. The SFM samples lost ~9.5% mass upon the first removal, while no appreciable silk weight loss occurred for the SFH group. After the first removal of loosened components at 24 h, all three groups showed no visible mass loss within 14 days.

After the first removal, the β-sheet content of SFL showed an increase from 15.8% to 21.2%, whereas the β-sheet content of SFM increased from 40.4% to 45.6% (Figs. 1b and 2c). At the end of immersion for 14 days, the β-sheet contents of SFL and SFM finally reached 22.8% and 47.6%, respectively (Fig. 2c). In contrast, the β-sheet content of SFH did not change evidently. The β-sheet contents of the three substrates remained significantly different between the groups during the whole immersion time.

The interfacial stability of FN was first measured by immersing the SF substrates in FN solution for 24 h followed by no treatment or the same ultrasonic treatment described above. Without the application of any artificial external forces, the FN was evenly distributed (Fig. 2d), and the fluorescence intensity of FN was comparable among all substrates (Fig. 2e). In contrast, when additional external forces were applied, an increase in dark areas (Fig. 2d), a lower fluorescence intensity of FN (Fig. 2e), and more detached FN (Supplementary Fig. 3a) were found on the SF substrates with a decreased β-sheet content. In addition, other alternative artificial external stimuli (e.g., Tris-EDTA buffer immersion) showed a similar trend in the detachment ratio of FN with the variation in β-sheet content (Supplementary Fig. 3b).

**Fig. 3:**
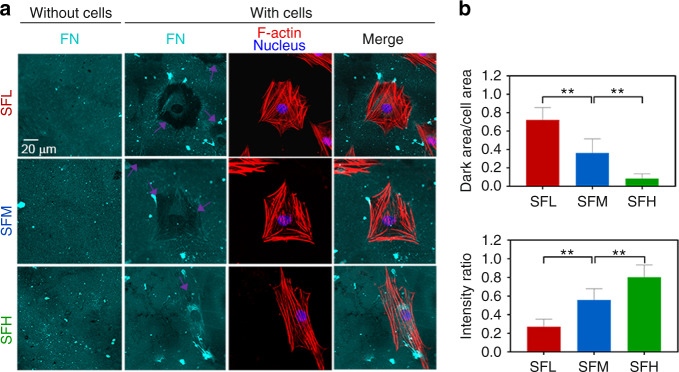
Interfacial stability of adsorbed FN on different substrates in a cell culture environment. **a** Immunofluorescence staining of adsorbed FN (cyan) on different SF substrates with and without BMSCs cultured for 24 h, together with F-actin (red) and nuclei (blue). The purple arrows indicate the detached FN underlying the cells. **b** Ratio of the FN dark area underlying the cell outline to the cell area. **c** Ratio of FN intensity underlying the cell outline to that of the background. Error bars represent one standard deviation. (*P < 0.05 and **P < 0.01)

To evaluate the interfacial stability of FN in a cell culture environment, we photographed the adsorbed FN and the morphology of BMSCs after 24 h of cell culture. The immunostaining images of FN and F-actin (Fig. 3a) and the subsequent analysis (Fig. 3b–c) showed an obvious FN dark area surrounding the cell outline on the SFL and SFM substrates, while the FN dark area was more evident on SFL than SFM. On the SFH substrates, the FN dark area was less apparent. The immunofluorescence staining images of FN on the substrates without BMSCs showed a relatively even distribution and no evident large dark area (Fig. 3a).

### Spreading behavior of BMSCs on different SF substrates

BMSCs were isolated, identified (Supplementary Fig. 4), and cultured on different SF substrates. The cell adhesion process within 4 h could be divided into four stages, and stage IV implied that cells adhered to the surface and appeared fully flattened and spread (Supplementary Fig. 5c).^19,35^ In this study, more cells were observed to enter stage IV on the SFH surfaces than on the SFM and SFL surfaces at 2 h and 4 h (Supplementary Fig. 5a, b), indicating a fast adhesion of BMSCs on surfaces with a high β-sheet content. However, the number of cells that successfully adhered to the surfaces was not significantly different among the groups (Supplementary Fig. 5d).

**Fig. 4:**
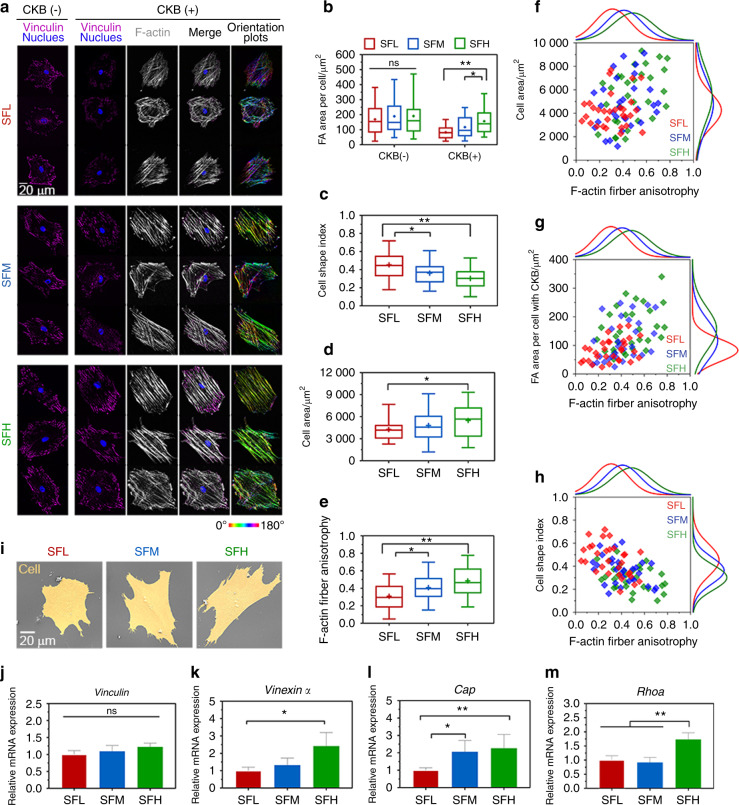
Spreading behavior of BMSCs on different SF substrates. **a** Representative mosaic immunofluorescence images of vinculin (purple), F-actin (white), and nuclei (blue) in BMSCs without and with CKB treatment and the corresponding orientation plots for F-actin staining, where the different colors indicate different orientations of actin filaments as per the given color map. **b–e** Quantitative analysis of (**b**) FA area per cell, (**c**) cell shape index, (**d**) cell area, and (**e**) F-actin anisotropy in BMSCs. **f–h** Single-cell scatter plots of F-actin anisotropy as a function of (**f**) cell area, (**g**) FA area per cell, and (**h**) cell shape index. **i** SEM images of BMSCs’ spreading morphology. **j–m** The mRNA expression levels of (**j**) *Vinculin*, (**k**) *Vinexin α*, (**l**) *Cap*, and (**m**) *Rhoa* in BMSCs. For each box plot, the box boundaries represent the 25th–75th percentiles, and the whiskers represent the min and max values. The central line and cross represent the median value and mean, respectively. Error bars represent one standard deviation. (*P < 0.05 and **P < 0.01)

After 24 h, BMSCs completed the process of adhesion and spreading onto the material surface.^19^ The immunostaining images of vinculin (Fig. 4a) and subsequent analysis (Fig. 4b) showed that the area of total vinculin-containing FAs per cell was comparable on all three SF-based interfaces without cytoskeletal buffer (CKB) treatment. However, after CKB treatment to remove proteins that are loosely attached to the cytoskeleton,^36^ the area of cytoskeleton-associated vinculin-containing FAs per cell was significantly larger on the SFH surfaces than on the SFM (*P* < 0.05) and SFL (*P* < 0.01) surfaces.

The immunostaining of F-actin (Fig. 4a) and the corresponding analysis (Fig. 4c, d) showed that the area of BMSC spreading was larger on the SFH surfaces than on the SFL surfaces. Cells exhibited significantly more elongated shapes (i.e., lower cell shape index (CSI)) on the SFH surfaces than on the SFM (*P* < 0.05) and SFL (*P* < 0.01) surfaces.

Furthermore, the corresponding orientation plots of F-actin showed that more actin stress fibers appeared to cluster into larger locally ordered microdomains aligned parallel to the long cell axis with the increase in the β-sheet content of the SF materials (Fig. 4a). Although such a large-scale order in cytoskeletal organization over the entire cell area was not observed at the SFL surfaces, some locally ordered actin filament microdomains were still observed. The quantitative analysis (Fig. 4e) of this F-actin fiber staining showed that the fiber anisotropy was significantly lower on the SFL surfaces than on the SFM (*P* < 0.05) and SFH (*P* < 0.01) surfaces.

Then, single-cell scatter plots showed F-actin fiber anisotropy as a function of cytoskeleton-associated FA area per cell or cell shape metrics (spread area and CSI). A clear trend was observed where a larger number of cytoskeleton-associated FAs was correlated with a higher-order of cytoskeletal organization, with distinct populations evident for the low, medium, and high β-sheet surfaces (Fig. 4g). In comparison, the scatter plots indicated a stronger correlation between F-actin fiber anisotropy and the CSI (Fig. 4h) than between F-actin fiber anisotropy and the cell area (Fig. 4f).

In addition, the SEM image depicted in Fig. 4i shows representative cell spreading morphology on different substrates. BMSCs were fully spread on all three surfaces, but cells revealed a more elongated shape on the SFH substrates than the SFL substrates. *Vinculin* expression in BMSCs showed no apparent differences among various SF surfaces (Fig. 4j), while *Vinexin α*, *Cap*, and *Rhoa* were upregulated on the SFH surface (Fig. 4k–m).

### Intracellular mechanotransduction of YAP/TAZ and RUNX2 on different SF substrates

**Fig. 5:**
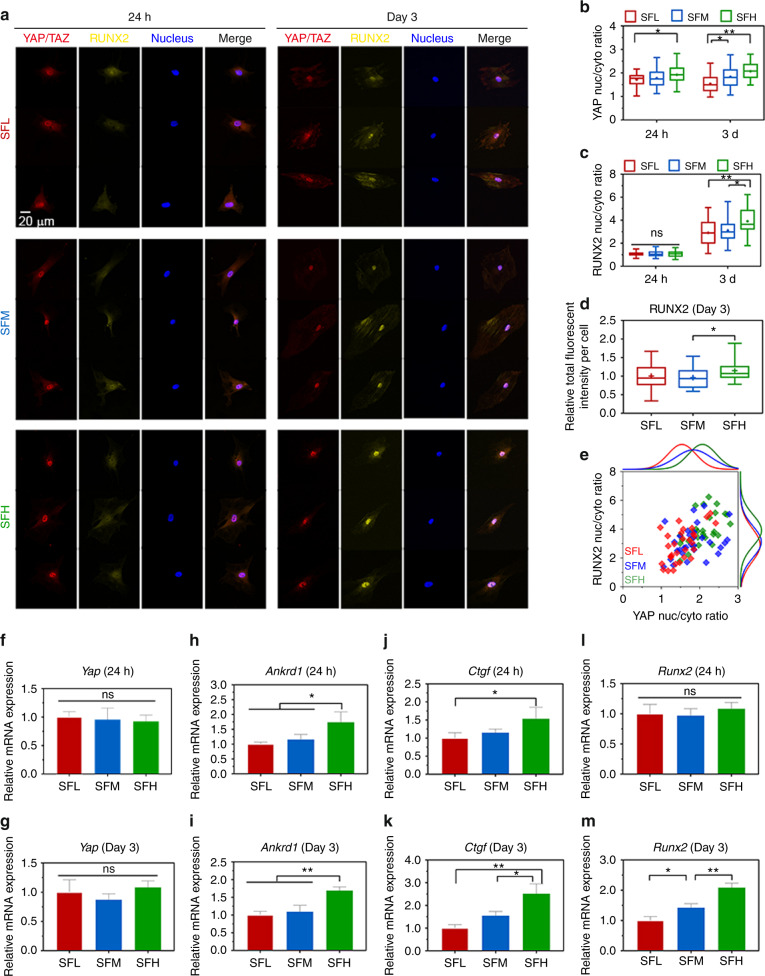
YAP/TAZ and RUNX2 nuclear translocation in BMSCs on different SF substrates. **a** Representative mosaic immunofluorescence images of YAP/TAZ (red), RUNX2 (yellow), and nuclei (blue) in BMSCs at 24 h and day 3. **b, c** The nuclear-cytoplasmic ratios of (**b**) YAP/TAZ and (**c**) RUNX2 in BMSCs analyzed from the immunofluorescence images. **d** Relative fluorescence intensity per cell of RUNX2 at day 3. **e** Single-cell scatter plots of the YAP/TAZ nuclei/cytoplasm ratio as a function of the RUNX2 nuclei/cytoplasm ratio at day 3. **f–m** The mRNA expression levels of (**f–g**) *Yap*, (**h–i**) *Ankrd1*, (**j–k**) *Ctgf*, and (**l–m**) *Runx2* in BMSCs at 24 h and day 3. For each box plot, the box boundaries represent the 25th–75th percentiles, and the whiskers represent the min and max values. The central line and cross represent the median value and mean, respectively. Error bars represent one standard deviation. (*P < 0.05 and **P < 0.01)

The immunostaining of YAP/TAZ in Fig. 5a shows that YAP/TAZ was translocated to the nucleus for all three groups, and YAP/TAZ was more abundant in the nuclei of BMSCs on the SFH surfaces than on the SFL surfaces at days 1 and 3. Quantitative analysis (Fig. 5b) revealed that the nuclear-cytoplasmic ratio of YAP/TAZ in BMSCs was lower on the SFL surfaces than on the SFH surfaces at day 1 and on the SFM (*P* < 0.05) and SFH (*P* < 0.01) surfaces at day 3.

Similarly, immunostaining of RUNX2 in BMSCs (Fig. 5a) indicated that RUNX2 nuclear translocation was not evident at day 1. In contrast, RUNX2 showed obvious nuclear translocation for all three substrates at day 3, and more RUNX2 was translocated from the cytoplasm to nucleus on SF substrates with an increase in β-sheets. The quantitative analysis (Fig. 5c) demonstrated that the nuclear-cytoplasmic ratio of RUNX2 in BMSCs was significantly higher on SFH vs. SFM (*P* < 0.05) and SFL surfaces (*P* < 0.01) at day 3, while it was comparable among all three SF surfaces at day 1. Moreover, quantitative measurement of the fluorescence intensity of RUNX2 per cell (Fig. 5d) indicated that RUNX2 expression in BMSCs was also higher on SFH than SFM at day 3.

The single-cell scatter plots (Fig. 5e) revealed a trend positively correlating the high nuclear ratio of YAP/TAZ with that of RUNX2, which indicates that RUNX2 might be nuclear translocated along with YAP/TAZ.

The expression of *Yap* in BMSCs was comparable for all different material surfaces at 24 h and day 3 (Fig. 5f, g). While the expression of *Ankrd1* and *Ctgf* was upregulated in the SFH samples compared to the SFL samples at 24 h and day 3 (Fig. 5h–k), *Runx2* expression was only upregulated with increased β-sheet content of the materials at day 3 (Fig. 5l, m).

When cytoskeletal organization was inhibited with the inhibitor Y27632, the difference in *Ankrd1* and *Runx2* gene expression in BMSCs on various SF surfaces was diminished at 24 h and day 3 (Supplementary Fig. 6), further confirming the relationship between cytoskeletal organization and intracellular mechanotransduction.

### Osteogenic differentiation of BMSCs on different SF substrates

**Fig. 6:**
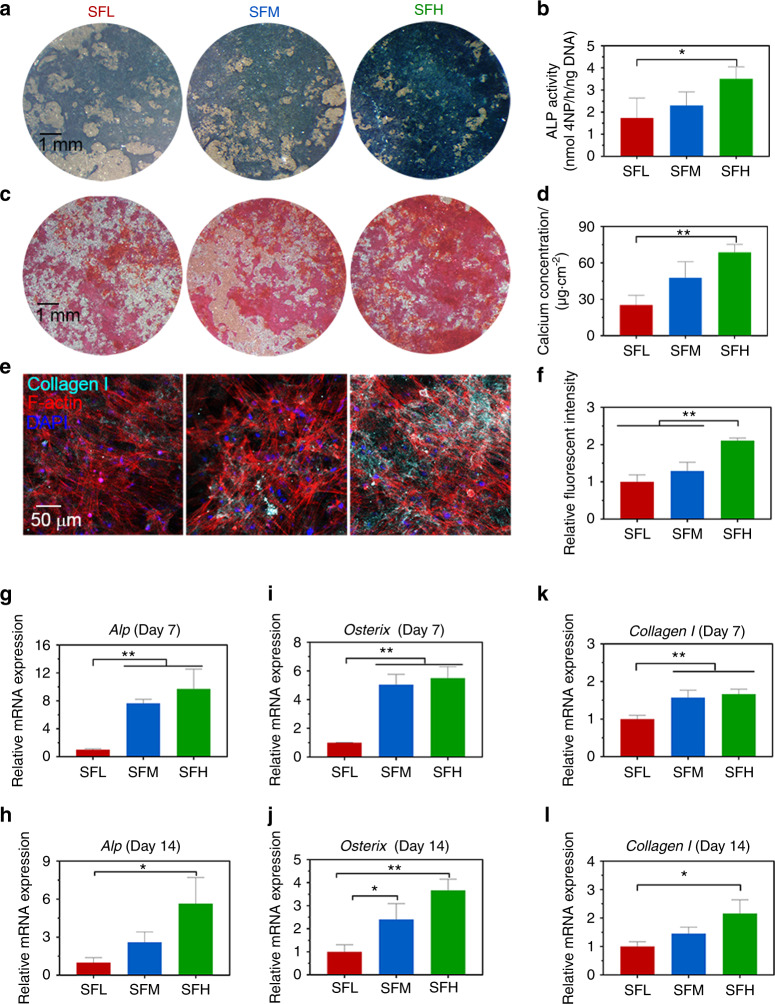
Osteogenic differentiation of BMSCs on different SF substrates. **a, b** Images of ALP staining (**a**) and quantitative detection of ALP activity (**b**) in BMSCs at day 7. **c, d** Images of ARS staining (**c**) and quantitative analysis of calcium concentration (**d**) in BMSCs at day 14. **e, f** Immunofluorescence images of collagen Ι (**e**) in BMSCs and quantitative analysis of fluorescence intensity of collagen Ι (**f**) at day 14. **g–l** mRNA expression levels of representative (**g–h**) *Alp*, (**i–j**) *Osterix*, and (**k–l**) *collagen Ι* in BMSCs at days 7 and 14. Error bars represent one standard deviation. (*P < 0.05 and **P < 0.01)

Alkaline phosphatase (ALP) expression was more abundant in the BMSCs with an increased β-sheet content of the SF materials at day 7 (Fig. 6a). Correspondingly, ALP activity was higher on the SFH vs. SFL surfaces (Fig. 6b). Different batches of BMSCs showed similar trends (Supplementary Fig. 7). The Alizarin staining images indicated that mineralization was also dependent on the β-sheet content of the SF materials at day 14 (Fig. 6c). More mineralization occurred on the SFH surfaces. Similarly, the quantitative detection kit showed that on the SFH surfaces, the concentration of calcium secreted by BMSCs was higher than that on the SFL surfaces (Fig. 6d). In addition, the immunostaining images (Fig. 6e) and their quantitative analysis (Fig. 6f) revealed that collagen Ι expression in BMSCs was upregulated on the SFH surfaces compared to the SFL and SFM surfaces at day 14.

The expression of the related osteogenic markers *Alp* (Fig. 6g, h), *Osterix* (Fig. 6i, j), and *Collagen Ι* (Fig. 6k, l) in BMSCs showed an upward trend on the SFH surfaces compared to the SFL surfaces at days 7 and 14. The cell number, as measured by the CCK-8 assay, was comparable on all SF surfaces (Supplementary Fig. 8).

## Discussion

The aim of the current study was to investigate the effect of the SF conformation on the osteogenic behavior of BMSCs. Herein, we prepared stiff SF substrates with different β-sheet contents. Depending on the various conformation conversion methods, the β-sheet contents in stiff SF materials range from ~15% to ~60% in the literature.^10–12^ Our prepared SF substrates contained three representative β-sheet contents within this range (Fig. 1b). The chemical composition of all materials was comparable since no additional chemicals except water were introduced during material preparation. Moreover, during these preparatory processes, the use of rigid titanium support successfully prevented obvious changes concerning surface topography and kept topography comparable within the microscopic scales (Fig. 1c), which may influence osteogenic cell behavior.^25^ With increasing β-sheet content, we found a slight (not significant) increase in Ra values (2.86–3.52 nm) (Fig. 1d). A previous study also observed a slight increase in the Ra values (2.12–3.28 nm) of SF films with an increase in β-sheet contents using graded ethanol (50%–100% v/v) treatments,^37^ but the low variation in nanoroughness did not significantly influence BMSC behavior.^37^

The range of water contact angles (60–70 degrees) of the SF substrates (Fig. 1e) was consistent with a previous study.^3^ This increase may result from the fact that the β-sheet formation induces tighter packing of hydrophobic repeats of the SF molecules, changing some amino acids exposed on the surface.^3^ However, these changes were subtle, and no significant difference was found. In addition, the one order of magnitude (14.8–120.3 MPa) in stiffness as a function of β-sheet content (Supplementary Table 1) was in accordance with the literature.^38^ Although the variation in stiffness within the Pa to kPa range can be sensed by cells and notably determines stem cell fate and osteogenic differentiation^25^, the variation in stiffness of 2D stiff material substrates for values higher than ~2 MPa does not significantly influence the osteogenic behavior of stem cells.^39,40^ Therefore, we do not expect that the stiffness of stiff SF substrates significantly determines the osteogenic cell behavior in this study.

Matrix metalloproteinases are the major enzymes secreted by BMSCs to degrade and remodel extracellular matrix and materials.^41^ However, Kaplan’s group demonstrated that the ability of matrix metalloproteinases to break down a solid SF film is not apparent, since the formation of SF-matrix metalloproteinase aggregates blocks degradation.^41^ Their in vitro study further confirmed that BMSCs do not induce significant endogenous enzymatic degradation of solid silk films as osteoclasts do.^5^ Therefore, to simplify the research model, we used a PBS solution instead of enzyme solution to investigate the material-protein interfacial stability of SF substrates in the aquatic environment.

Before the formation of β-sheets, SF materials are water soluble and mainly consist of SF molecules presenting random coil conformations.^17^ The conformation transition results in soluble random coil SF molecules folding into a stable β-sheet conformation to form water-insoluble molecule networks, in which the remaining random coil regions and other soluble protein fragments are embedded.^12^ When the amount of β-sheet exceeds a threshold (~15%), the network is dense enough to make the bulk SF material water insoluble.^15^ Our SEM images (Fig. 2a) revealed that some soluble fractions can still easily dissolve and escape from the relatively loose β-sheet network of an SFL substrate under external forces, although the bulk material is water-insoluble. This finding indicates the surface instability of the material with a low β-sheet content.

This observation was confirmed by the quantification of the remaining mass of SF substrates subjected to the same artificial external force treatment (Fig. 2b). The 29.2% total weight loss of SFL substrate is close to the reported ~35% total weight loss of a low β-sheet content (~20%) silk film, which is prepared by the slow-drying method and tested via a degradation/dissolution experiment in PBS without external force.^12^ Accordingly, another PBS degradation/dissolution study reported a total weight loss (~17%) of a medium β-sheet content (~30%) film induced by water annealing for 6 h at room temperature,^17^ which is close to the observed 14.5% total weight loss of the SFM substrates.

Another notable finding is that after the first 24 h immersion and ultrasonic treatment, the SFL and SFM substrates already showed an initial weight loss of 24.5% and 9.5%, respectively, whereas the substrates exhibited no further visible (>5%) mass loss during the 14 days of immersion. This finding indicates that except for the loosened components, the SF molecule network was stable and could maintain the structural integrity of the SF material. Notably, the effect of the surface instability of stiff SF materials on cells will be different compared with the influence of extensively investigated hydrogel degradation characteristics on cells. Hydrogels undergo sustained surface erosion and bulk degradation due to high water content and diffusivity, which progressively breaks the structural integrity of materials and can favor BMSC spreading and osteogenic differentiation by reducing the steric hindrance effect.^28,42^

The stability of adsorbed proteins on materials was also assessed by immersion of the SF substrates in FN solution for 24 h followed by no treatment or the same artificial external forces above. Without ultrasonic treatment, the fluorescence intensity of FN was comparable among all three substrates (Fig. 2e), which is not surprising considering the fact that the morphology and wettability of the substrates were comparable.^31^ However, with ultrasonic treatment, a larger dark area and lower fluorescence intensity of FN were observed on the SF surfaces with decreased β-sheet content. This finding suggests that more adsorbed FN was detached with the removal of the loosened components from the low β-sheet content material surfaces by the application of the external force.^22^ These results indicate that a high content of the β-sheets stabilized the protein-material interface, and a low content of the β-sheets might compromise the detachment resistance of adsorbed FN via the reduced surface stability of the SF materials. A similar influence of material surface properties on the detachment resistance of FN was reported in previous studies, e.g., the long-chain collapse of a material surface was shown to induce the detachment of FN.^43^

These different phenomena of detachment resistance of adsorbed FN on various SF substrates were further investigated by culturing cells on top of this protein-coated surface (Fig. 3). The immunofluorescence images of FN on substrates without cell culture showed a relatively even distribution without dark areas, indicating that the observed dark area underlying BMSCs was caused by the presence of cells.^22^ Moreover, our results showed more obvious dark areas on the SFL substrates than on the SFH substrates, which indicates that more FN was detached by the transition of cellular force to the adsorbed FN layer on the SFL surface. In addition, the degree of FN detachment induced by cells showed similar degrees of FN detachment resistance as that observed in PBS with artificial external force treatment. Once cells adhere, part of the FN will desorb during the attachment phase. Simultaneously, cells will also generate tensions via their cytoskeleton and exert cell contractility on the adsorbed protein layer of underlying materials through FAs,^25^ which might induce our observation of partial FN detachment from the SF surfaces.^22^

Furthermore, the influence of protein/material interfacial stability on BMSC behavior was studied. Among various proteins that form FAs, vinculin plays a crucial role in FA assembly and actin polymerization.^20^ The structure of vinculin can switch between activated (linked to F-actin) and inactivated (head–tail folding structure inhibiting its association with F-actin).^36,44^ Immunostaining revealed an increase in cytoskeleton-associated (i.e., activated) vinculin in BMSCs cultured on the SFH surface compared to SFL (Fig. 4b). This result is consistent with the observed upregulated expression of *Vinexin α* and *Cap* in the BMSCs cultured on the SFH surface (Fig. 4k, l), which is related to the activation and unfolding of vinculin.^36,45^

The cellular assays also demonstrated that the BMSCs exhibited a more elongated shape (Fig. 4c) with a higher degree of cytoskeletal organization on SFH surfaces than on SFL surfaces (Fig. 4e). The emergence of cellular scale order in cytoskeletal organization has also been observed on substrates when other types of mechanobiological stimuli were applied, e.g., different geometric micropatterns^46^ and frequent mechanical stress.^47^ Moreover, the anisotropy value (0.485) of F-actin fibers in the BMSCs on SFH is close to the reported data (~0.5) found in BMSCs cultured on typically stiff and stable material surfaces (e.g., glass),^46^ implying that the order of cytoskeletal organization on SFH reached its upper limit.

Single-cell scatter plots showed two trends: (i) a larger number of cytoskeleton-associated FAs of the BMSCs on higher β-sheet content surfaces along with higher-order cytoskeletal organization (Fig. 4g) and (ii) a higher-order cytoskeletal organization correlated with more elongated cell shapes (Fig. 4h). This finding is supported by the upregulated gene expression of *Rhoa* in the BMSCs cultured on the SFH surfaces (Fig. 4m). Previous studies demonstrated that via exposure of its cryptic binding sites to link F-actin, unfolded vinculin can trigger a series of phosphorylation events to activate the mechanoresponsive signaling transforming protein, RhoA, which engages in the control of cytoskeleton dynamics and promotes cell elongation and polarity.^21,44^

Thus, a lower stability of the adsorbed protein-material interface was found along with a smaller number of cytoskeleton-associated FAs, lower order in cytoskeleton organization, and less elongated cell shapes in the BMSCs cultured on the SFL surfaces compared to the SFH surfaces. These observations can be explained by the tensegrity model, which shows that cytoskeletal ordering and cell polarity require high resistance to endogenous stress.^23^ The instability of the adsorbed protein-material interface of substrates with low β-sheet content may partially reduce the intracellular tension and stress resistance by the adsorbed protein-FA-cytoskeleton link.^22^ Compared to the cellular effect induced by low stiffness, the spreading cell shape and cytoskeletal organization pattern observed here in the BMSCs on stiff but unstable SFL surfaces are completely different from those on soft materials. On a soft material (e.g., hydrogel) surface, most cells present a round and nonspreading shape with an orthoradial pattern of actin filaments around the nucleus.^19,35^

In this intracellular mechanotransduction process, the nuclear-cytoplasmic ratio of YAP/TAZ was higher on the SFH surface than on the SFL surface (Fig. 5b). In the nuclei, YAP/TAZ are activated and can interact with and activate a number of their DNA binding partners (e.g., RUNX2^30–32^) to modulate the osteogenic differentiation of stem cells. This higher nuclear-cytoplasmic ratio was correlated with the higher gene expression levels of *Ankrd1* and *Ctgf* in the BMSCs cultured on SFH than in those cultured on SFL (Fig. 5h–k). *Ankrd1* and *Ctgf* are YAP/TAZ target genes, and their expression patterns are used to monitor the activity of YAP/TAZ.^24^

After 3 days, higher gene (Fig. 5m)/protein (Fig. 5d) expression and nuclear localization of RUNX2 (Fig. 5c) were also observed in the BMSCs on the SFH surfaces than the SFL surfaces. The single-cell scatter plots (Fig. 5e) indicate that RUNX2 and YAP/TAZ might undergo nuclear translocation together. The YAP/TAZ complex can promote BMSC osteogenic differentiation by enhancing RUNX2-dependent transcriptional activation.^24,48,49^ This material surface-dependent nuclear cotranslocation and activation of YAP/TAZ and RUNX2 in BMSCs has also been reported on material substrates engineered by other mechanical stimulations (e.g., nanotopography).^31^ To confirm this effect of intracellular mechanotransduction on osteogenic differentiation, we further cultured BMSCs on different silk material surfaces for 2 weeks. The results showed an increased osteogenic differentiation of BMSCs on the SFH surface than on the SFL surface (Fig. 6). The upregulated expression of *Osterix* (Fig. 6i, j), a downstream gene of RUNX2,^16^ on the SFH surfaces, is consistent with the observation that RUNX2 was activated more effectively on that surface.

**Fig. 7:**
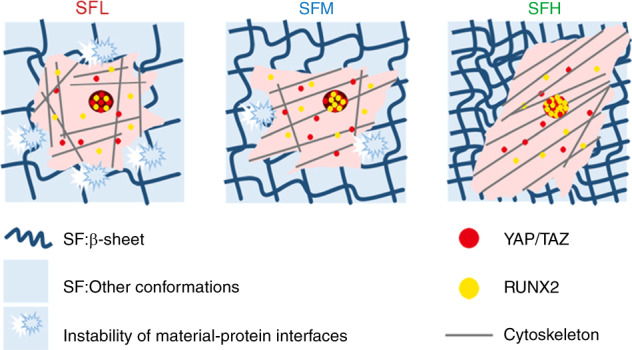
The molecular conformation of SF regulates osteogenic cell behavior by modulating the stability of the adsorbed protein-material interface. Applying FN as a model cellular adhesive protein, the surface stability of the SF substrates and the accompanying detachment resistance of adsorbed protein on these SF substrates increased with increasing β-sheet content. Furthermore, more cytoskeleton-associated FAs, higher orders of cytoskeletal organization, and more elongated spreading shapes were observed in the BMSCs cultured on the SFH surface than in those cultured on the SFL surface, along with the enhancement of nuclear translocation and activation of YAP/TAZ and RUNX2 in intracellular mechanotransduction

Although many studies have focused on osteogenic signaling and regulating the osteogenic potential of stiff SF materials via their physical (e.g., topography designs), chemical (e.g., decorated chemical groups) and biological (e.g., immobilized growth factors) factors,^50^ few have revealed the impact of SF molecular conformation on osteogenic cell behavior. Using FN as a model cellular adhesive protein, this study found that the surface stability of the SF substrates and the accompanying detachment resistance of adsorbed protein showed a positive correlation with the β-sheet content. Moreover, we observed (i) more cytoskeleton-associated FAs, (ii) higher-order cytoskeletal organization, and (iii) more elongated cell spreading for BMSCs cultured on the SF substrates with high vs. low β-sheet content, along with enhanced nuclear translocation and activation of YAP/TAZ and RUNX2. Consequently, osteogenic differentiation of BMSCs was stimulated on high β-sheet substrates. These results indicate that the β-sheet content may influence the osteogenic differentiation of BMSCs on stiff SF material surfaces by modulating the detachment resistance of adsorbed protein, which proceeds via protein-FA-cytoskeleton-YAP/TAZ-RUNX2 mechanotransduction (Fig. 7).

Moreover, by using stiff SF substrates as an example, we found that the stability of the protein-material interface can play an important role in the cellular perception of the stiff material interface. The unstable material surface and accompanying inadequate detachment resistance of adsorbed cellular adhesive protein may partially impede the mechanotransduction of cells anchoring onto the material surface. This phenomenon should not be overlooked when engineering stiff biomaterial (e.g., silk) interfaces for bone-related applications. However, how this stability of the protein-material interface regulates BMSC behavior on stiff material surfaces in 3D, long-term, and multifactorial in vivo conditions still needs to be investigated further.

## Materials and methods

### Construction of SF substrates with different conformations

*B. mori* silk cocoons were first degummed in boiled 0.02 mol·L^−1^ Na_2_CO_3_ solution for 30 min and then washed with Milli-Q water. After drying, the extracted silk was dissolved in 9.3 mol·L^−1^ LiBr solution at 60 °C for 4 h and then dialyzed with Milli-Q water.^10^ Insoluble residues were removed by centrifugation. Finally, the SF aqueous solution was diluted to 4 wt%.

To facilitate good handling and avoid morphological changes of SF materials during the experiments, we prepared silk substrates by casting 200 μL of SF solution onto titanium disks (15 mm diameter). To enhance the contact area and adhesion of SF film with titanium disk and avoid detachment of SF film during the whole experiments, we first acid etched the titanium disks with HCl/H_2_SO_4_ for 30 min at 60 °C, ultrasonically cleaned them in acetone, ethanol and water, and finally subjected them to argon plasma glow discharge (Radio frequency glow discharge machine, Harrick Scientific Corp., U.S.A.) for 5 min before the SF solution casting. The SF films were dried in a vacuum oven to avoid structural changes.^38^ Finally, the films were annealed in a water vapor-filled vacuum chamber at 4 °C for 6 h (SFL group), 37 °C for 12 h (SFM group), or 90 °C for 24 h (SFH group) to achieve different β-sheet contents in the SF substrates. The thickness of the SF films made by this method was ~50 μm.^10,38^

### Fourier transform infrared spectroscopy (FTIR)

FTIR analysis of the silk films was performed using attenuated total reflectance infrared spectroscopy (UATR Two, PerkinElmer, the Netherlands). For calculation of the β-sheet content in the different SF substrates, the contribution of the different SF conformations to the amide I region (1 595–1 705 cm^−1^) was determined by Fourier self-deconvolution using PerkinElmer software and subsequent curve fitting by OriginPro software according to a previously reported step-by-step method (*n* = 3).^15,51^

### Surface topography of the SF substrates observed via SEM

The surface topography of SF substrates dried in a vacuum oven was examined by SEM (Zeiss, Sigma-300, Germany) after being coated with a 10 nm chromium layer.

### AFM

The surface topography and roughness of the SF substrates were measured using AFM (Bruker, multimode 8, U.S.A.) (*n* = 3, three random points per sample). The stiffness of the SF films was measured by using AFM cantilevers (SNL-10, Bruker, multimode 8, U.S.A.) with a nominal spring constant of 0.35 N·m^−1^. Samples for stiffness measurement were first hydrated with PBS solution,^38^ and the force vs. indentation curves were obtained in PBS on each SF substrate. Elastic modulus values were analyzed by NanoScope Analysis software.

### Wettability

The wettability of different SF substrates was determined by detecting the static water contact angles of the surfaces with an optical tensiometer (Theta Lite Attension®, Biolin Scientific, Sweden) (*n* = 3).

### Surface stability of the SF substrates

For detection of the surface stability in an aqueous environment, samples were immersed in 2 mL of PBS solution at 37 °C for 14 days. PBS was refreshed every 24 h. At specified time points, a mild ultrasonic treatment was applied to remove any loosened components from the material surfaces before removing the samples from PBS. The probe of the sonicator (UP50H, Hielscher, Germany) was located 1 cm right above the sample, and mild ultrasonic treatment lasted for 6 s at 20% amplitude with a pulse rate of 1 s on and 1 s off according to previous references.^41^ Then, the samples were gently rinsed with Milli-Q water and dried in a vacuum oven. The remaining mass of SF materials at specified time points was calculated using the following formula:

$${{{{Remaining}}\,{{mass}}}}\,{{\% \,=\, }}\frac{{{{W_t}}}}{{{{W_0}}}}{\it{ \times 100\% }},$$

where *W*_*0*_ represents the initial weight of the sample and *W*_*t*_ represents the weight of the sample at a specified time point (*n* = 3).

### Detachment resistance of FN

FN (5 μg·mL^−1^) from human plasma (F1056, Sigma, U.S.A.) was dissolved in PBS solution according to previous references.^19,22^ The SF substrates were immersed in FN solution for 24 h. Then, the samples were treated without or with the same ultrasonic treatment used in the surface stability experiments before removing the samples from the solution. Subsequently, all samples were incubated with the corresponding primary and secondary antibodies (Supplementary Table 2) to fluorescently label FN. Images were captured by a fluorescence microscope (Axio Imager Microscope Z1, Zeiss, Germany), and fluorescence density was analyzed by ImageJ (*n* = 3, three random fields per sample).

For determination of the FN detachment ratio under external stimuli, FN concentrations in solutions were determined by a FN Human ELISA kit (BMS2028, Invitrogen, U.S.A.). The detachment ratio was calculated using the following formula:

$${{{{FN}}\,{{detachment}}}}\,{{\% \,=\, }}\frac{{{{C_2}}}}{{{{C_0 - C_1}}}}{{ \times 100\% }},$$

where *C*_0_ represents the initial concentration of FN added to the PBS solution, *C*_1_ represents the concentration of FN remaining in the PBS solution after 24 h of immersion, and *C*_2_ represents the concentration of FN detached under external stimuli (*n* = 3).

In addition, immersion of samples in 50 mmol·L^−1^ Tris buffer pH 7.4 containing 1 mmol·L^−1^ EDTA for 24 h at 37 °C^52^ was chosen as an alternative artificial external stimulus for reference.

### Cell isolation and identification

BMSCs were isolated from the femurs of 3-week-old male rats (Charles River) with the approval of the Institutional Animal Care and Use Committee of Tongji Medical College (IACUC Number: 539). For each batch, primary cells derived from at least five rats were pooled together, and three batches of cells were made to verify the reproducibility of the experiments. Cells were cultured in growth medium consisting of α-MEM medium (Gibco, Invitrogen Corp., Paisley, Scotland) supplemented with 10% fetal bovine serum (FBS, Gibco, Invitrogen Corp., Paisley, Scotland) and 1% penicillin-streptomycin according to a standard protocol.^40,53^ The pluripotency of BMSCs was identified with a flow cytometer (LSRFortessa, BD, U.S.A.). Cells were harvested and suspended to a concentration of 1 × 10^6^ cells per mL in ice-cold PBS, and CD90, CD44, and CD29 were used as positive markers, while CD31 was used as a negative marker (Supplementary Table 3). Cells were passaged to the 3rd generation at a confluency of 70%–80% before use.

### Cell culture

Untreated SF substrates were sterilized by ultraviolet light for 30 min. For analysis of the detachment resistance of FN in a cell culture environment, the SF substrates were first immersed in serum-free (to avoid the interference of FN from serum) cell culture medium (α-MEM) containing 5 μg·mL^−1^ FN for 2 h.^19,22^ Then, BMSCs were seeded at a density of 2 × 10^3^ cells per cm^2^ in serum-free medium and cultured for 24 h, while SF substrates immersed in the same medium without cells were used as controls.

For other cell assays, untreated SF substrates were first immersed in complete osteogenic medium (α-MEM, 10% FBS, 50 μg·mL^−1^ ascorbic acid (A4544, Sigma, U.S.A.), 10 mmol·L^−1^ β-glycerophosphate (G9422, Sigma, U.S.A.), 10^−8^ M dexamethasone (D4902, Sigma, U.S.A.), and 1% penicillin-streptomycin) for 2 h before cell seeding. Then, cells were seeded in this complete osteogenic medium. For the individual cell-based fluorescent measurements, a seeding density of 2 × 10^3^ cells per cm^2^ was applied for the cell adhesion, spreading, and intracellular mechanotransduction experiments according to the literature.^32^ For the fluorescent staining of RUNX2 and YAP/TAZ at day 3, the SF substrates were treated with mitomycin C (10 μg·mL^−1^, Sigma, U.S.A.) for 2 h after 12 h of cell seeding to inhibit proliferation, which can interfere with their individual cell-based fluorescence measurements.^32^ In addition, 1 × 10^4^ cells per cm^2^ BMSCs were seeded to assess cell proliferation and osteogenic differentiation.

### Immunofluorescence staining

At specific experimental time points, cells were fixed with 4% paraformaldehyde and permeated with 0.1% Triton X-100. Subsequently, the cells were blocked with 1% goat serum for 30 min, incubated with the corresponding primary and secondary antibodies (Supplementary Table 3) and incubated for 1 h. Samples were incubated with phalloidin for 30 min and DAPI for 10 min to label F-actin and mark cell nuclei, respectively.

CKB treatment could remove the proteins that were not associated with the cytoskeleton. CKB treatment was applied as described previously to distinguish the amount of cytoskeleton-associated vinculin-containing FAs from the total amount of vinculin-containing FAs.^36^ Briefly, to observe the cytoskeleton-associated (i.e., CKB treatment-resistant) vinculin-containing FAs, cells were first treated twice with CKB (0.1% Triton X-100, 10 mmol·L^−1^ PIPES, pH 6.8, 50 mmol·L^−1^ NaCl, 3 mmol·L^−1^ MgCl_2_, 300 mmol·L^−1^ sucrose) at 4 °C for 30 s, followed by a standard fixation step with 4% paraformaldehyde. Images of stained samples were captured by confocal fluorescence microscopy (Zeiss, LSM780, Germany).

### Fluorescent image analysis

All images were analyzed by ImageJ (NIH, U.S.A.).

For collagen Ι quantification, the mean intensity of four random fields across three samples per group was measured.

FN detachment induced by cell contractility was measured using a method as previously described.^22^ In brief, the cellular masks were determined by the thresholding method from F-actin fluorescent images and used to delineate the cell outline. We calculated the ratio of the dark area underlying the cell outline in FN staining images to the total cell area. In addition, we calculated the ratio of FN intensity underlying the cell outline to the background (the other area beyond the cell outline) FN intensity. Four random fields across three samples per group were measured.

At least 30 individual cells across three samples per group were selected for all cell-based measurements. For measurement of the FA area per cell, the grayscale vinculin image was thresholded to produce a black and white image from which the pixels representing FAs were counted and summed, following a step-by-step quantitative FA analysis protocol as previously reported.^54^

For measurement of cell area and perimeter, the cellular masks were determined by a thresholding method from F-actin fluorescent images, and then, they were used to calculate cell area and cell perimeter. The CSI was calculated using the formula as previously reported:^28^

$${\mathrm{{{CSI}}}} \,=\, \frac{{{\it{4\uppi }} \times {{cell}}\,{{area}}}}{{{{cell}}\,{{perimeter}}^{\it{2}}}},$$

where a line and a circle have CSI values of 0 and 1, respectively.

F-actin anisotropy was measured by the ImageJ plug-in “FibrilTool” as previously reported,^55^ where disordered F-actin (purely isotropic fibers) and perfectly ordered F-actin (parallel fibers) have anisotropy values of 0 and 1, respectively.

The nuclear/cytosolic (nuc/cyto) ratio of YAP/TAZ and RUNX2 was determined by measuring the intensity of a region of the nucleus and a region of equal size in the cytosol immediately adjacent to the nuclear region as previously described.^36^

### Cell spreading observed by SEM

Samples were fixed in 2% glutaraldehyde for 20 min and then immersed in 0.1 mol·L^−1^ Na-cacodylate for 10 min. Then, the samples were dehydrated in a graded series of ethanol (5 min in 70%, 80%, 90%, 96%, and 10 min in 100% ethanol twice). The samples were then treated with 1 drop of tetramethylsilane, air-dried, coated with 10 nm chromium, and examined via SEM.

### ALP and mineralization

After 7 days of cell culture, ALP activity in BMSCs was measured using the p-nitrophenyl phosphate (Sigma, U.S.A.) method as previously described.^19^ The ALP activity was normalized by DNA content per sample via a QuantiFluor dsDNA System kit (E2670, Promega Corporation, Madison, U.S.A.). For ALP staining, cells were fixed and stained with an ALP Staining Kit (AP100B-1, SBI, U.S.A.), followed by taking images via a stereomicroscope (MZ12, Leica, Germany). After 14 days, calcium contents were assayed using the ortho-cresolphthalein complexone (Sigma, U.S.A.) method as previously described.^48^ For mineralization staining, cells were fixed and then stained with an Alizarin Red S staining kit (TMS-008-C, Merck Millipore, U.S.A.), followed by taking images via stereomicroscope.

### Quantitative reverse transcription polymerase chain reaction (qRT-PCR)

The RNA of BMSCs on different silk substrates (*n* = 3) was extracted using the RNAprep Pure Micro Kit (DP420, Tiangen, China) according to the manufacturer’s instructions. The RNA was reverse transcribed to cDNA using HiScript III RT SuperMix for qPCR (R323-01, Vazyme, China). Subsequently, cDNA was added to ChamQ SYBR qPCR Master Mix (Q311-02/03, Vazyme, China) and complemented by a real-time PCR system (ABI 7300, Applied Biosystems, U.S.A.). The primers for the genes are listed in Supplementary Table 4. The mRNA levels of target genes were normalized to the level of GAPDH mRNA and calculated via the 2^-ΔΔCt^ method.

### Inhibition of cytoskeleton organization

For analysis of the inhibition of cell spreading and cytoskeletal organization, Y27632 (50 μmol·L^−1^, Selleck, U.S.A.) was supplemented daily with the cell culture medium, and the treatment lasted 4 h prior to cell harvesting as previously described^24^ for fluorescent staining and qRT-PCR.

### Cell proliferation

Cell proliferation on different material surfaces (*n* = 3) was assessed by a cell counting kit (CCK-8, Dojindo, Japan) according to the manufacturer’s instructions.

### Statistical analysis

One-way ANOVA was used to determine statistical significance followed by post hoc analysis using the Tukey test. All statistical analyses were performed with GraphPad Prism and Origin software.

## Supplementary information

Supplementary information-revised
