# Supplementary material for: The molecular conformation of silk fibroin regulates osteogenic cell behavior by modulating the stability of the adsorbed protein-material interface
Source: Bone Res. 2021 Feb 11;9:13. doi: 10.1038/s41413-020-00130-0 (PMC7878842; doi:10.1038/s41413-020-00130-0)
Supplement: Supplementary file 1 — Supplementary information-revised [file 41413_2020_130_MOESM1_ESM.docx]

Supplementary Information

Molecular Conformation of Silk Fibroin Regulates Osteogenic Cell Behavior by Modulating the Stability of Adsorbed Protein-Material Interface

Yanlin Long, ^‡, a,b^ Xian Cheng,^‡,c^ John A. Jansen,^c^ Sander G.C. Leeuwenburgh,^c^ Jing Mao, ^d^ Fang Yang,*^,c^ Lili Chen*^,a,b^

^a^Department of Stomatology, Union Hospital, Tongji Medical College, Huazhong University of Science and Technology, Wuhan 430022, China.

^b^Hubei Province Key Laboratory of Oral and Maxillofacial Development and Regeneration, Wuhan 430022, China.

^c^Department of Dentistry – Biomaterials, Radboud University Medical Center, Philips van Leydenlaan 25, 6525 EX Nijmegen, The Netherlands

^d^Center of Stomatology, Tongji Hospital, Tongji Medical College, Huazhong University of Science and Technology, Wuhan 430022, China.

‡YL and XC contributed equally to this work.

* Corresponding authors:

Lili Chen : Tel: +86-27-85726949; Fax: +86-27-85726949;

Fang Yang: Tel: +31 627892931; Fax: +31 627892931


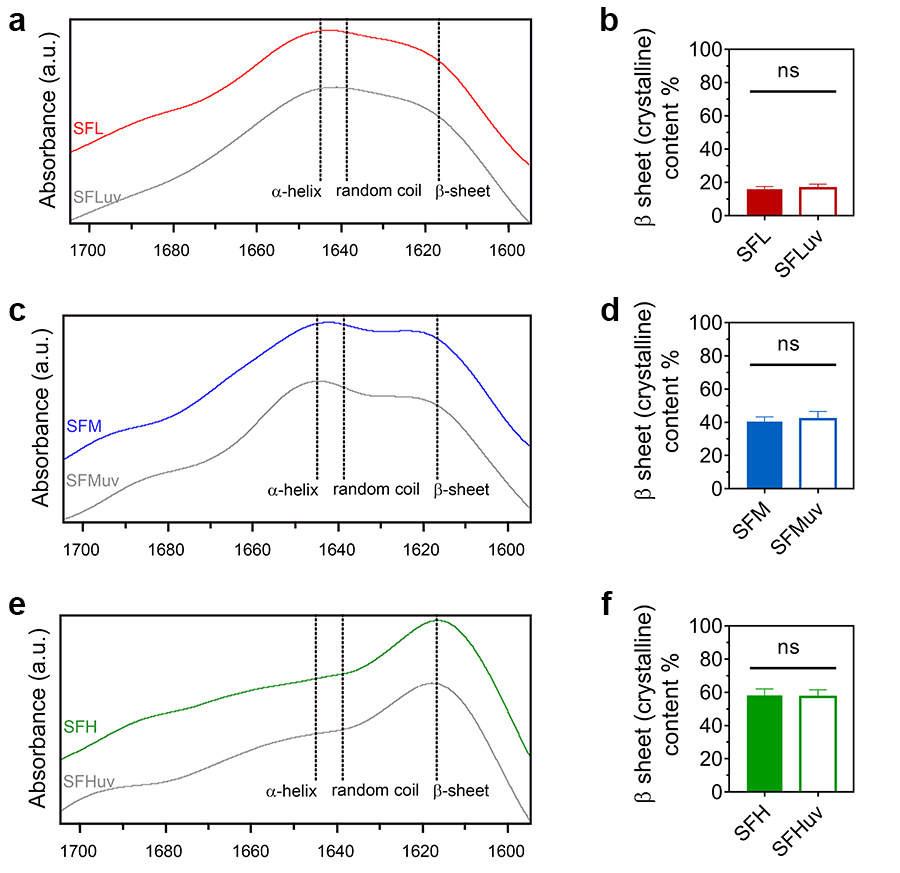
**Supplementary Figure 1.** The influence of ultraviolet sterilization on the conformation of different SF substrates. **a-f** FTIR absorbance spectra (**a, c ,e**) of amide I region (between 1695 and 1595 cm^−1^) obtained from different silk materials before and after ultraviolet sterilization and the corresponding β-sheet content (**b, d, f**) calculated by Fourier self-deconvolution. The data from Figure 1a and b were displayed here as the groups before ultraviolet sterilization for comparison. (*p < 0.05 and **p < 0.01).


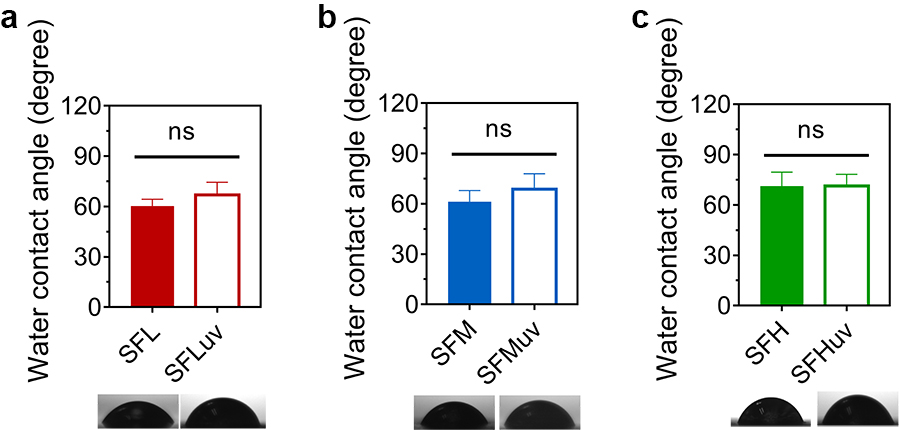


**Supplementary Figure 2.** The influence of ultraviolet sterilization on the wettability of different SF substrates. **a-c** the surface wettability measured by the water contact angle test, and the representative images of water droplets on different SF substrates before and after ultraviolet sterilization. The data from Figure 1e and f were displayed here as the groups before ultraviolet sterilization for comparison. Error bars represent one standard deviation. (*p < 0.05 and **p < 0.01).


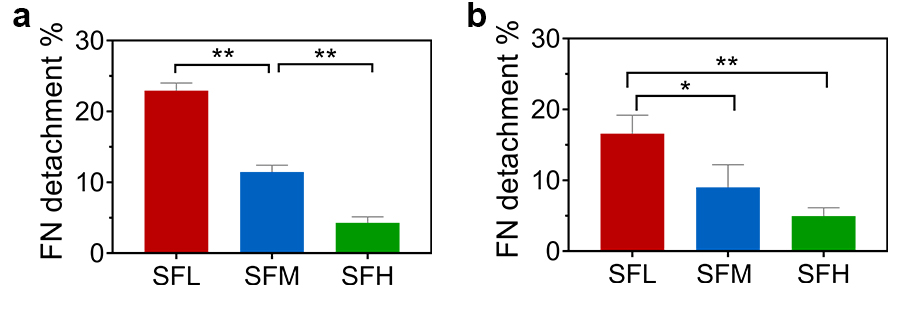


**Supplementary Figure 3.** The detachment ratio of FN on different SF substrates. **a, b** The detachment ratio of FN under (**a**) ultrasonic treatment or (**b**) Tris-EDTA buffer immersion. Error bars represent one standard deviation. (*p < 0.05 and **p < 0.01).

**
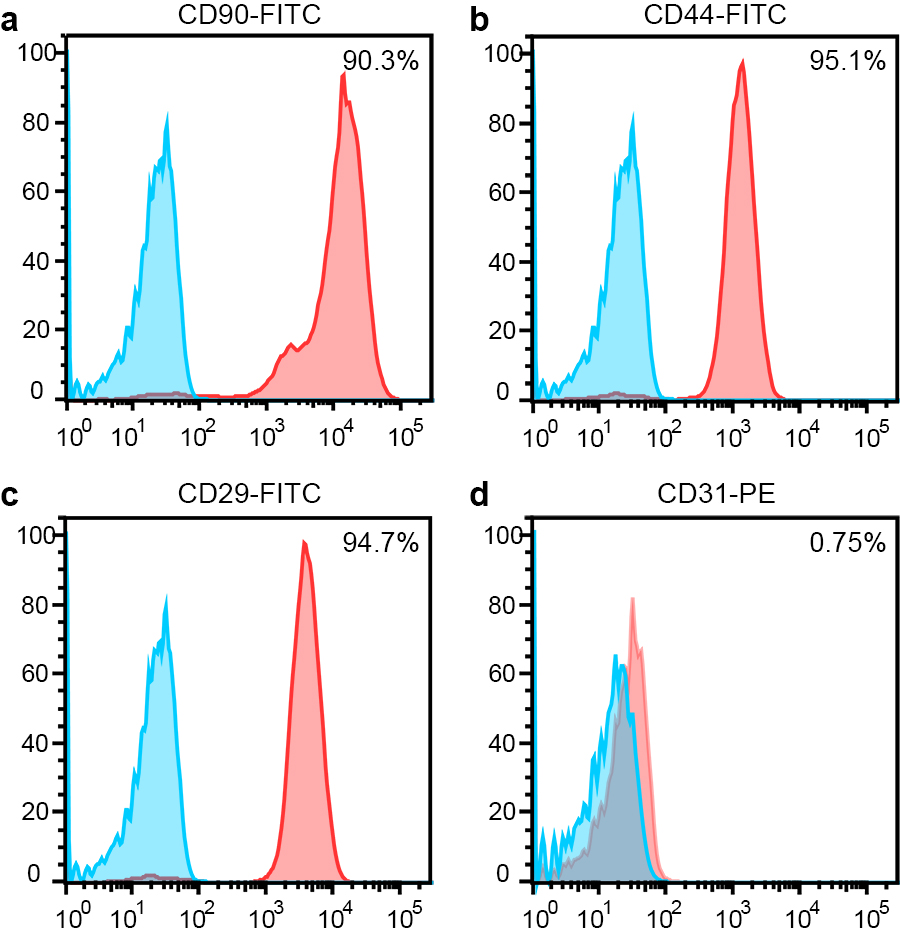
**

**Supplementary Figure 4.** The pluripotency of BMSCs identified with flow cytometry. **a-d** Expression of positive index of (**a**) CD90, (**b**) CD44, and (**c**) CD29 in cells. Expression of negative index of (**d**) CD31 in cells.

**
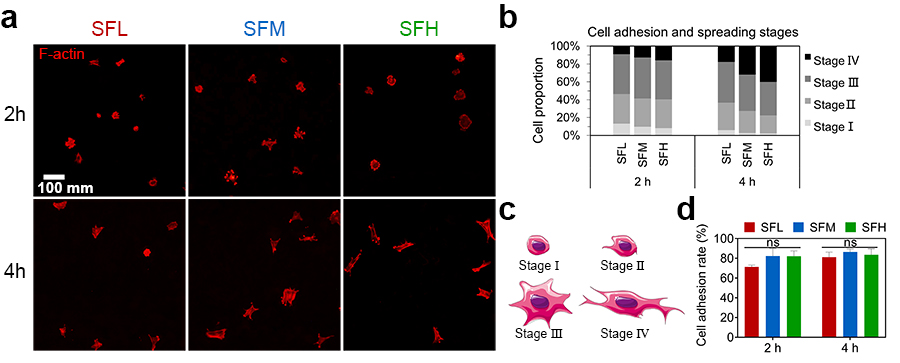
**

**Supplementary Figure 5.** Adhesion process of BMSCs on different SF substrates. **a** Immunofluorescence images of cell adhesion and spreading morphology. F-actin (red), and nucleus (blue). **b** Proportion of cell adhesion stages on different SF material surfaces at 2 h and 4 h. **c** Typical schematic diagrams of cell adhesion morphology of four stages. **d** Cell adhesion rate of MSC on different silk materials. Error bars represent one standard deviation. (*p < 0.05 and **p < 0.01).


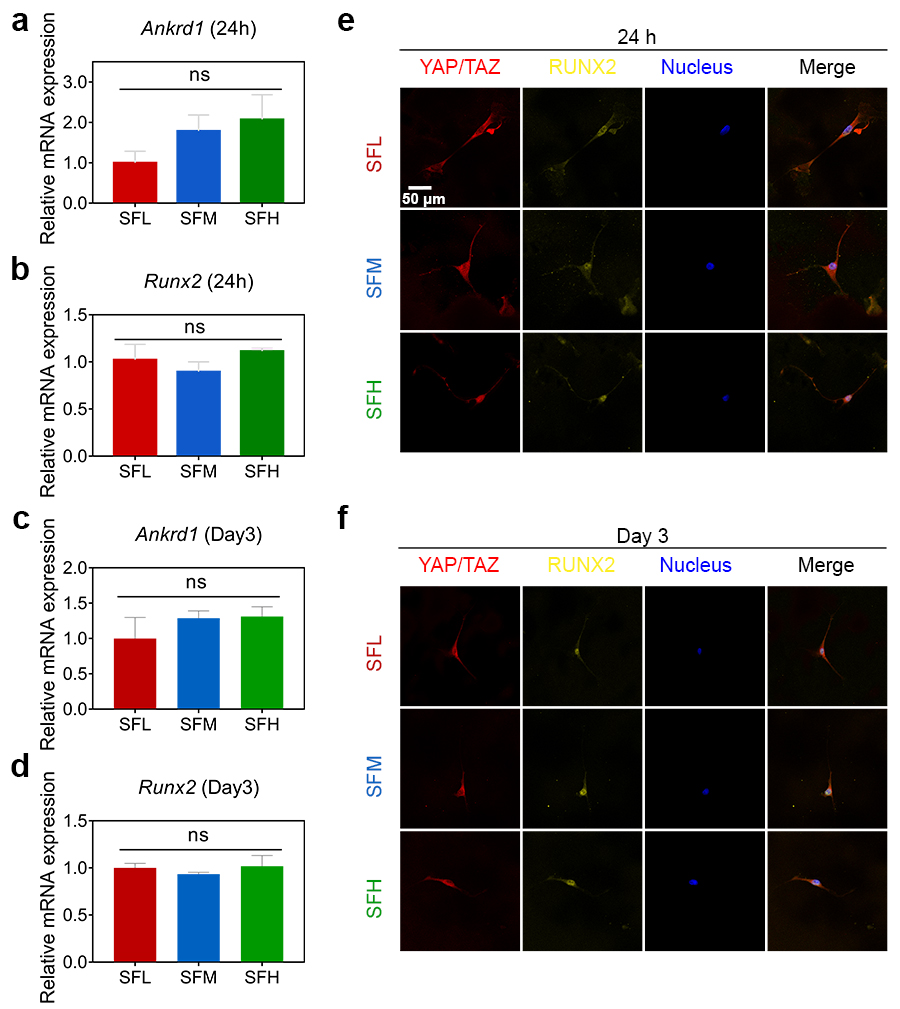


**Supplementary Figure 6.** Inhibiting the cytoskeletal organization in BMSC on different SF substrates with inhibitor Y27632. **a-d** The mRNA expression levels of *Ankrd1* and *Runx2* in MSCs with Y27632 treatment after 24 hours and 3 days. **e-f** Immunofluorescence images of YAP/TAZ (red), RUNX2 (yellow), and nucleus (blue) in MSCs with Y27632 treatment after 24 hours (**e**) and 3 days (**f**). Error bars represent one standard deviation. (*p < 0.05 and **p < 0.01).

**
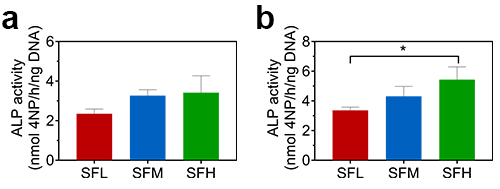
**

**Supplementary Figure 7.** ALP activity detection with different batches of MSCs. **a, b** ALP activity of batch 2 (**a**), batch 3 (**b**). Error bars represent one standard deviation. (*p < 0.05 and **p < 0.01).

**
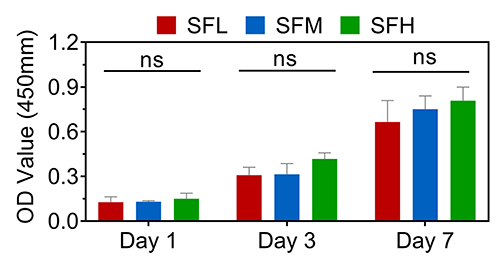
**

**Supplementary Figure 8.** BMSC proliferation on different SF substrates. CCK-8 analysis at day 1, 3, and 7. Error bars represent one standard deviation. (*p < 0.05 and **p < 0.01).

**Supplementary Table 1.** **Surface stiffness of SF materials with different conformations.**

| Groups | Mean  (MPa) | SD (MPa) |
| --- | --- | --- |
| SFL | 14.8 | 4.1 |
| SFM | 62.5 | 20.4 |
| SFH | 120.3 | 17.4 |

**Supplementary Table 2. Antibodies used in flow cytometry.**

| Antibodies | No. | dilutions | Brand |
| --- | --- | --- | --- |
| FITC-Anti-CD90 | #202503 | 1:200 | BioLegend, U.S.A. |
| FITC-Anti-CD44 | #203906 | 1:200 | BioLegend, U.S.A. |
| FITC-Anti-CD29 | #102205 | 1:50 | BioLegend, U.S.A. |
| PE-Anti-CD31 | 555027 | 1:200 | BD,U.S.A. |

**Supplementary Table 3. Antibodies used in immunofluorescence.**

| Antibodies | No. | dilutions | Brand |
| --- | --- | --- | --- |
| Fibronectin (without cells) |  |  |  |
| Rabbit Anti-Fibronectin | ab2413 | 1:200 | Abcam, U.K. |
| Goat Anti-Rabbit IgG H&L (Alexa-Fluor 594) | ab150080 | 1:800 | Abcam,U.K. |
| Fibronectin (with cells) |  |  |  |
| Rabbit Anti-Fibronectin | ab2413 | 1:200 | Abcam, U.K. |
| Goat Anti-Rabbit IgG H&L (Alexa-Fluor 647) | ab150083 | 1:500 | Abcam,U.K. |
| Collagen 1 |  |  |  |
| Rabbit Anti-Collagen 1 | ab34710 | 1:500 | Abcam,U.K. |
| Goat Anti-Rabbit IgG H&L (Alexa-Fluor 647) | ab150083 | 1:500 | Abcam,U.K. |
| Vinculin |  |  |  |
| Rabbit Anti-Vinculin | ab129002 | 1:200 | Abcam, U.K. |
| Goat Anti-Rabbit IgG H&L (Alexa-Fluor 647) | ab150083 | 1:500 | Abcam,U.K. |
| YAP/TAZ |  |  |  |
| Mouse Anti-YAP/TAZ | sc101199 | 1:50 | Santa Cruz,U.S.A. |
| Goat Anti-Mouse IgG H&L (Alexa-Fluor 594) | ab150116 | 1:500 | Abcam,U.K. |
| RUNX2 |  |  |  |
| Rabbit Anti-RUNX2 | ab192256 | 1:500 | Abcam,U.K. |
| Goat Anti-Rabbit IgG H&L (Alexa-Fluor 647) | ab150083 | 1:500 | Abcam,U.K. |
| F-actin |  |  |  |
| TRITC-phalloidin | P1951 | 1:2000 | Sigma, U.S.A. |
| Nucleus |  |  |  |
| DAPI | D9542 | 1:2500 | Sigma, U.S.A. |

**Supplementary Table 4. Gene primers used in qRT-PCR.**

| Gene | Forward primers | Reverse primers | Species |
| --- | --- | --- | --- |
| *Gapdh* | AGGGCTGCCTTCTCTTGTGAC | ATCTCGCTCCTGGAAGATGGTG | Rat |
| *Ctgf* | ACCCAACTATGATGCGAGCCA | CCGGATGCACTTTTTGCCCTT | Rat |
| *Ankrd1* | AAGGTGCCAAAATCAGTGCCC | TAGCGATTCAACCTCACCGCA | Rat |
| *Osterix* | AGTTCACCTGTCTGCTCTGCT | GCGGCTGATTGGCTTCTTCTT | Rat |
| *Vinexin α* | TGAGAAACCTGCACAGCCCAT | GTCGTGGAGCCTGAGAGTTCTT | Rat |
| *Cap* | ACATTGACGGGGAGAAAGTGGT | TTGGGCTCTGCTCGGTATTTTC | Rat |
| *Yap* | AGACGCTGATGAACTCTGCCT | ATGGCAAAACGAGGGTCAAGC | Rat |
| *Vinculin* | AGCACCCAGCTCAAAATCCTGT | AGAGTAAATCCGGCGTCTGTTC | Rat |
| *Rhoa* | GTGTTTTTCCATCGACAGCCCT | TCTTCAGGTTTTACCGGCTCCT | Rat |
| *Runx2* | ATGGTACTTCGTCAGCGTCCT | AGACTCATCCATTCTGCCGCT | Rat |
| *Col1* | CTGCCCAGAAGAATATGTATCACC | GAAGCAAAGTTTCCTCCAAGACC | Rat |
| *Alp* | CGTCTCCATGGTGGATTATGCT | CCCAGGCACAGTGGTCAAG | Rat |
